# Factors associated with avoidable hospitalisation of children younger than 2 years old: the 2006 Brazilian National Demographic Health Survey

**DOI:** 10.1186/s12939-015-0204-9

**Published:** 2015-08-21

**Authors:** Tulio Konstantyner, Laís Amaral Mais, José A. A. C. Taddei

**Affiliations:** Department of Health Sciences, University of Santo Amaro (UNISA), Sao Paulo, Brazil; Department of Paediatrics, Discipline of Nutrology, Federal University of São Paulo (UNIFESP), Rua Loefgreen, 1647, CEP: 04040-032 São Paulo, SP Brazil

## Abstract

**Introduction:**

Ambulatory Care Sensitive Conditions (ACSC) are conditions for which hospitalisation is thought to be avoidable with the use of effective preventive care and early disease management. The objective of this study was to estimate the rate of avoidable hospitalisations in children younger than 24 months of age participating in a Brazilian national representative survey and to identify the risk factors for such hospitalisations.

**Methods:**

We analysed data from a cross-sectional study of 1901 children from the 2006 Brazilian National Demographic Health Survey of Women and Children (NDHS). The children’s socioeconomic, biological and maternal characteristics, nutritional status, and access to healthcare were tested; variables with *p* < 0.20 were selected to fit a Poisson regression.

**Results:**

The prevalence of avoidable hospitalisation was 11.8 % (95 % Confidence Interval [CI], 9.0, 15.2); the prevalence was higher in the Southeast (40.1 %) and Northwest (21.7 %) macro-regions. The multivariate model identified five risk factors for avoidable hospitalisation: male gender (Prevalence Ratio [PR] = 1.48, *p* = 0.004), low socioeconomic level (PR = 1.51, *p* = 0.005), children from mothers younger than 20 years of age (PR = 1.41, *p* = 0.031), not breastfed within the first hour of life (PR = 1.29, *p* = 0.034), and neonatal hospitalisation (PR = 1.66, *p* = 0.043).

**Conclusions:**

To decrease the costs associated with avoidable hospitalisations, health managers and professionals should focus their efforts on providing effective primary healthcare to families of low socioeconomic levels, particularly prenatal and paediatric care, as well as encouraging breastfeeding and supporting young mothers. Strategies to improve children’s health by controlling such hospitalisations in Brazil should consider all residence areas and geopolitical macro-regions.

## Introduction

Ambulatory Care Sensitive Conditions (ACSC), also known as Potentially Avoidable Paediatric Hospitalisations (PAPH) when related to children and adolescents, are conditions for which hospitalisation is thought to be avoidable with the application of effective preventive care and early disease management, which is usually delivered in a primary care setting. It is an indicator that correlates with access, quality, effectiveness, and use of primary health care [1–5].

Avoidable hospitalisation rates have been used as quality indicators of healthcare provided to populations, as access indicators of primary care, and as indicators of outpatient care related to primary care capacity. These indicators, based on the fact that appropriate use of ambulatory care services can reduce hospitalisation, can help to expand policies and programs related to morbimortality, which can decrease the costs of second and third level health care and improve patient quality of life by decreasing patient suffering [1, 4, 6–10].

Infants have the greatest risk of being hospitalised for ACSC, particularly in developing countries. This result can be explained by their immature development and specific health conditions related to physical growth and neurophychomotor development, resulting in a physiological vulnerability for illness [8, 11, 12]. Focusing in early childhood years is beneficial because it is the opportune time to intervene and prevent the long-term consequences of poor health [13].

The Brazilian National Health System (SUS) intents to provide equity in health by ensuring universal and free access to primary care, although there are still a lot of inequalities between regions. Since 1990, a Brazilian primary healthcare policy initiative (i.e., the Family Health Strategy), which focuses on community primary health care as a way to guarantee access, coverage and continuity, has decreased avoidable hospitalisation rates in children by performing systematic follow-ups and ensuring health care accessibility [5, 8, 14].

Diarrhoea and respiratory diseases, particularly pneumonia, are the primary causes of avoidable hospitalisation in Brazilian infants. According to the Brazilian Ministry of Health, because of ACSC, hospitalisation rates decreased by 4 % in children younger than 1 year of age between 2002 and 2012 (9.2 % vs. 5.2 %, respectively) [12, 15, 16].

Generally, paediatric hospitalisations should only occur if there is no safer outpatient treatment for the disease because children placed in unfamiliar environments can experience psychological consequences. In addition, because hospital care is responsible for a significant amount of health investments, the reduction of avoidable hospitalisations would directly benefit health system planning [1, 12].

Since Brazil is a developing country with a large territorial area and great socioeconomic and cultural diversity, the regions’ differences can express peculiar epidemiological characteristics of infants’ avoidable hospitalisation. Understanding inequalities in health related to access and equity issues, such as socioeconomic and demographic characteristics, is important to help health professionals identify vulnerable groups and develop strategies for control and prevention of ACSC, aiding early diagnosis, reducing the rate of related diseases and allocating resources to improve paediatric healthcare, especially to Brazil and other countries with similar inequities and demography [5, 13, 17].

Globally, the most important risk factors related to avoidable hospitalisation rates in infancy are black skin colour, male gender, low maternal educational level, low family income, low maternal age, living in an urban area, higher family density, low birth weight, early weaning, tobacco smoking, cold and humidity exposure, and difficulty accessing outpatient care [1, 6, 11–13].

The aim of this study was to estimate the rate of avoidable hospitalisations and to identify the associated risk factors in infants from the “*Pesquisa Nacional de Demografia e Saúde da Criança e da Mulher*” (the Brazilian National Demographic Health Survey of Women and Children [NDHS—2006]), a representative demographic health survey conducted by the Brazilian Ministry of Health [15].

## Methods

Data from the NDHS—2006 were used in this study. The NDHS aimed to describe the demographic profiles of Brazilian women of childbearing age and children younger than 5 years of age. This research is part of the MEASURE Demographic and Health Survey (DHS), an investigation globally conducted and supported by United States Agency for International Development (USAID) and several other international institutions, which aims to provide data and analyses of health indicators in developing countries. In Brazil, the NDHS—2006 is the third and last edition of this research, which is performed in every 10 years [15].

The NDHS—2006 is a nationally representative household sample obtained using a selection performed in two stages: primary units comprised of census tracts, and secondary units formed by households. A probabilistic distribution including the five Brazilian regions and urban and rural areas was used. The NDHS—2006 selected sample units The sample design, data collection standards and procedures, data consistency, expansion technique for the complex sample and ethical issues have been reported elsewhere [15].

We analysed data from children younger than 24 months of age who participated in the NDHS—2006. The initial sample consisted of 1902 infants. One participant was excluded from the study due to a lack of information regarding previous hospitalisations. Additionally, 75 children were excluded from the multivariate analysis due to a lack of data on the variables included in the model. Therefore, data from 1901 children were considered in the bivariate analysis, and data from 1826 children were included in the multivariate analysis of avoidable hospitalisations.

The participants whose mothers reported that they had any avoidable hospitalisation within the last 12 months were included in the outcomes analysis. Only one outcome was studied: avoidable hospitalisation. According to the ‘Brazilian List of ACSC’, we considered as avoidable hospitalisation any hospitalisation for the following reasons (diseases): diarrhoea, pneumonia, bronchitis, and others that potentially are avoidable paediatric hospitalisations by an appropriate ambulatory health care (e.g. infectious diseases and nutrition disorders) [18]. The Prevalence Ratios (PR) and factors associated with avoidable hospitalisation were calculated, taking into account the complex NDHS—2006 sampling design, as mentioned above [2, 15].

In choosing the variables, we considered factors that have been identified in the literature as being associated with avoidable hospitalisation, including specific characteristics of the mother and children (e.g. maternal and child’s age, maternal education and smoking, child’s sex, weight at birth and nutritional status), the children’s access to health services and family socioeconomic conditions. Children’s nutritional status were analysed using z-score for height for age, weight for age, and weight for height, as recommended by the World Health Organization (WHO), and all the index were calculated using the WHO Anthro software [19, 20]. To analyse family income, we used a per capita income based on the minimum wage for the year in which the data were collected (monthly minimum wage in 2006 = US$163.00). The socioeconomic classes of the families were estimated using the “*Associação Brasileira de Empresas de Pesquisa*” (Brazilian Association of Research Companies [ABEP]) level (Brazilian socioeconomic criteria). It consists in a score system, which is calculated for every residence by assessing the total value of household goods and the education level of the head of household. This socioeconomic criteria has been used in Brazilian health researches and it is considered the most appropriate tool to be used to evaluate the purchasing power of families in representative samples [21].

To estimate the hospitalisation prevalence rates in Brazil by cause and by macro-regions (descriptive analyses), we used the established criteria by the original sample design, which were performed with the expansion technique that provided national representativeness for five Brazilian macro-regions (North, Northeast, Midwest, South and Southeast) and two residence areas (urban and rural), and considering the sample weight for both primary and secondary units [15].

To describe the sample population, we presented their characteristics as continuous and categorical variables. To investigate associations and estimate prevalence ratios (bivariate and multivariate analyses), we used dichotomous variables to perform a Poisson analysis, considering just the expansion technique [22, 23]. The cut-off points for the dichotomous variables were based on recommendations of official agencies or were obtained through a literature review of the topic [19, 24].

To adjust for confounding factors, a Poisson multivariate analysis was performed using a forward stepwise technique. The selection criteria for the explanatory and control variables for inclusion in the final model were those associations with avoidable hospitalisation corresponding to a *p* < 0.20 [25]. A maximum level of *p* = 0.05 was chosen to indicate a significant association and, thus, was retained in the final model. Three controlling variables that conceptually interfered with avoidable hospitalisation rates were retained in the final model: child age, residence area, and macro-region.

The Stata statistical software package, version 12.0 [26] was used to analyse the data and to expand the sample.

## Results

**Fig. 1:**
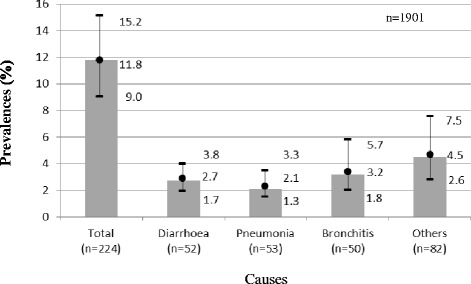
Hospitalisation prevalence (%) by cause for children younger than 2 years old (Brazil, NDHS, 2006)

We found that 224 Brazilian infants were hospitalised by ACSC in the 12 months preceding the 2006 survey, which equals 237 hospitalisations due to the occurrence of more than one admission per child occasionally. Those numbers resulted in a prevalence rate of 11.8 % hospitalisations countrywide (95 % Confidence Interval [CI], 9.0, 15.2). The hospitalisation prevalences were similar by cause, varying between 2.1 % and 4.5 %. The highest prevalence was related to respiratory diseases (pneumonia and bronchitis) resulting in 5.3 %, almost a half of the total hospitalisation rate (Fig. 1).

**Fig. 2:**
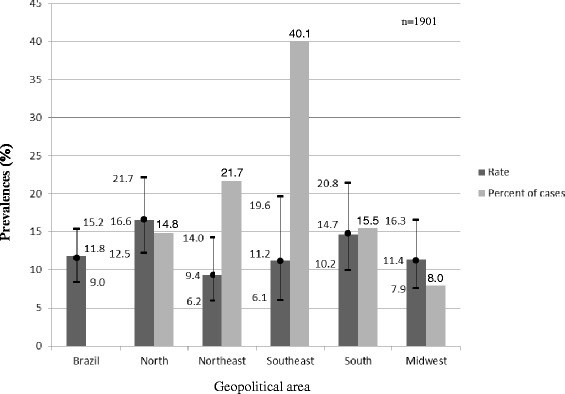
Hospitalisation prevalence (%) of children younger than 2 years old (Brazil and macro-regions, NDHS, 2006)

When categorised by the five Brazilian macro-regions, the highest avoidable hospitalisation rates occurred in the North (16.6 %) (95 % CI, 12.5, 21.7) and South (14.7 %) (95 % CI, 10.2, 20.8) macro-regions. However, the greatest percentages of hospitalisations occurred in the Southeast (40.1 %), followed by the Northeast (21.7 %). Although the Southeast hospitalisation rate (11.2 %) was not one of the highest, four in each ten children in Brazil were hospitalised in this macro-region (Fig. 2).

Table 1 reports the prevalence estimates and means of the infant characteristics. The mean children’s and mother’s age were 11.0 (95 % CI, 10.5, 11.6) and 25.4 (95 % CI, 24.9, 26.0), respectively. The mean *per capita* family income (US$162.35) was lower than the Brazilian minimum wage for 2006 (US$163.00). The percentage of male children was 52.6 % (95 % CI, 48.2, 56.9). Only 39.1 % of the infants were breastfed in the first hour of life, and the mean number of exclusive breastfeeding days was 101.3. Besides, 5.4 % (95 % CI, 3.0, 7.8) of children were hospitalised during the first days of their life (neonatal hospitalisation).

**Table 1:** Averages and prevalences of the characteristics of children younger than 2 years old (Brazil, NDHS, 2006)

Characteristics (continuous variables)	*n*	μ	(95 % CI)	Characteristics (categorical variables)	*n*	P (%)	(CI 95 %)
Age (months)	1901	11.0	(10.5;11.6)	Urban residence area	1901	82.3	(77.6;86.9)
Mother’s age (years)	1901	25.4	(24.9;26.0)	Mother’s age younger than 20 years	1901	18.7	(15.0;22.4)
Maternal education (years)	1894	7.9	(7.6;8.2)	Maternal education <8 years	1901	38.0	(33.3;42.7)
People in household	1901	4.4	(4.2;4.5)	Sex (male)	1901	52.6	(48.2;56.9)
Siblings in household	1074	1.5	(1.4;1.6)	ABEP level (D-E)	1872	61.9	(56.7;67.2)
Per capita family income (R$)	1662	248.6	(212.7;284.6)	Macro-region (S and SE)	1901	54.4	(49.2;59.6)
Prenatal (visits)	1803	8.0	(7.6;8.3)	Neonatal hospitalisation	1850	5.4	(3.0;7.8)
Weight at birth (g)	1860	3250	(3213;3295)	Breastfeeding in the first hour of life	1901	39.1	(34.7;43.6)
Exclusive breastfeeding (days)	1851	101.3	(95.8;106.8)	Maternal smoking	1901	14.0	(10.8;17.3)
Height for age^a^	1752	−0.15	(−0.29; −0.02)	Primiparous mother	1901	48.9	(44.4;53.4)
Weight for age^a^	1805	0.16	(0.08;0.26)	Lack of sanitation	1901	76.2	(72.0;80.5)
Weight for height^a^	1737	0.33	(0.23;0.44)	Lack of maternal marital stability	1901	13.6	(10.0;17.2)

*μ* mean, *P* prevalence, *CI* confidence interval, *ABEP level* Brazilian socioeconomic criteria, *R$* Real (Brazilian currency), *S* South, *SE* Southeast

^a^z-score

The independent variables and their associations with avoidable hospitalisation are presented in Table 2. In the bivariate analysis, the variables related to socioeconomic characteristics, children’s health care conditions, biological aspects, maternal characteristics, and children’s nutritional status were associated with children’s hospitalisations. Among these variables, the child’s age (*p* < 0.001), sex (*p* = 0.004), and ABEP level (*p* = 0.006) demonstrated the strongest associations. Besides, sanitation conditions, breastfeeding in the first hour of life, neonatal hospitalisation, maternal education (years), marital status, and height for age (z-score) were significantly associated with avoidable hospitalisation. As shown in Table 2, the variables that reported *p* < 0.20 were eligible for adjustment in the multivariate analysis.

**Table 2:** Prevalence ratio of hospitalisations and characteristics of children younger than 2 years old (NDHS, 2006)

Variables (*n*)	*N*	Categories	Hospitalisations n (%)		PR	*p*
Socioeconomic characteristics						
Residence area	1901	Urban	1284	12.41 (156)	1.10	0.496
		Rural	617	8.70 (68)	1.00	
ABEP level	1872	D-E	1209	12.56 (159)	1.48	0.006
		A-B-C	663	10.05 (59)	1.00	
Sanitation conditions	1901	Inadequate	1590	14.07 (198)	1.49	0.043
		Adequate	311	4.31 (26)	1.00	
People in household	1901	≥4	1405	13.68 (177)	1.33	0.073
		<4	496	7.96 (47)	1.00	
Per capita family income (minimum wage)	1901	≤1	1420	13.32 (174)	1.18	0.287
		>1	481	7.42 (50)	1.00	
Child healthcare conditions						
Exclusive breastfeeding (months)	1901	<6	1440	10.88 (158)	0.77	0.051
		≥6	461	14.24 (66)	1.00	
Breastfeeding in the first hour of life	1901	No	733	13.30 (102)	1.33	0.019
		Yes	1168	10.75 (122)	1.00	
Neonatal hospitalisation	1850	Yes	90	26.58 (18)	1.72	0.015
		No	1760	10.97 (204)	1.00	
Prenatal visits	1901	<6	398	16.22 (52)	1.14	0.388
		≥6	1503	10.80 (172)	1.00	
Biological aspects						
Age (months)	1901	<12	980	8.17 (75)	0.47	<0.001
		≥12	921	15.85 (149)	1.00	
Birthweight (Kg)	1901	<2.5	118	20.08 (17)	1.24	0.365
		≥2.5	1783	11.28 (207)	1.00	
Sex	1901	Male	1002	14.53 (139)	1.47	0.004
		Female	899	8.67 (85)	1.00	
Maternal characteristics						
Education (years)	1901	<8	835	13.99 (114)	1.32	0.027
		≥8	1066	10.37 (110)	1.00	
Marital status	1901	Without partner	283	22.84 (43)	1.36	0.049
		With partner	1618	10.00 (181)	1.00	
Age (years)	1901	<20	283	20.49 (41)	1.28	0.117
		≥20	1618	9.73 (183)	1.00	
Smoker	1901	Yes	248	21.02 (36)	1.28	0.142
		No	1653	10.24 (188)	1.00	
Primiparous	1901	Yes	780	12.12 (86)	0.89	0.392
		No	1121	11.40 (138)	1.00	
Child nutritional status						
Height for age (z-score)	1901	<−2	159	28.22 (28)	1.57	0.015
		≥ − 2	1742	10.35 (196)	1.00	
Weight for height (z-score)	1901	<−2	49	16.08 (8)	1.40	0.363
		≥ − 2	1852	11.62 (216)	1.00	
Weight for age (z-score)	1901	<−2	61	13.43 (8)	1.12	0.735
		≥ − 2	1840	11.70 (216)	1.00	

*PR* prevalence ratio, *ABEP level* Brazilian socioeconomic criteria

**Fig. 3:**
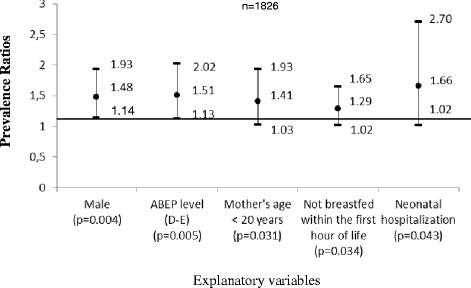
Adjusted prevalence ratio (PR) of hospitalisations of children younger than 2 years old (Brazil, NDHS, 2006). Adjusted model for child age, residence area and macro-region

Figure 3 demonstrates the prevalence ratio that remained in the final multiple model (*p* < 0.05). Male children (PR = 1.48, *p* = 0.004) from the lowest ABEP level (PR = 1.51, *p* = 0.005), with mothers younger than 20 years of age (PR = 1.41, *p* = 0.031), who were not breastfed in the first hours of life (PR = 1.29, *p* = 0.034), and who had been hospitalised immediately after birth (PR = 1.66, *p* = 0.043) independently demonstrated a greater risk of avoidable hospitalisation, when adjusted for child age, residence area and macro-region.

## Discussion

The results of this study indicated that 11.8 % of children younger than 24 months were hospitalised in the past 12 months, particularly because of diarrhoeal and respiratory diseases. The highest avoidable hospitalisation rate occurred in the North, and the highest percentage of cases occurred in the Southeast. Overall, the hospitalisations were independently associated with male gender, lower socioeconomic level, younger mothers, no breastfeeding in the first hour of life, and neonatal hospitalisation.

Although paediatric hospitalisations because of ACSC have occurred less frequently in the last decades in Brazil, high rates still occur in the five macro-regions, despite being avoidable, as demonstrated by the results of this study [8, 27]. Globally, the rate of ACSC in paediatric groups has decreased with age, meaning that the rates decrease after 2 years of age [2, 4]. However, a comparison of available studies showed that these rates vary greatly due to wide variability among the conditions considered to be sensitive enough for ambulatory care [1, 6, 28].

Diarrhoea, pneumonia and bronchitis were the main causes of hospitalisation of Brazilian children in this study. This finding is consistent with those reported by national and international research studies [29–32]. Diarrhoea results from a lack of access to piped water, low sanitation conditions, low family income, and low maternal education. Reduced diarrhoea rates have been observed due to the expansion of oral rehydration therapy use; however, it is still considered to be a public health problem worldwide, particularly among developing countries and in children younger than 5 years of age [27, 33, 34].

However, pneumonia and bronchitis are responsible for a large percentage of hospitalisations among children younger than 5 years old worldwide. These diseases are related to poor air conditions, exposure to smoke, household overcrowding and low family income [33, 35, 36].

Those respiratory diseases can be influenced by many socioeconomic and environmental factors; therefore, the rates of avoidable hospitalisation are sensitive to regional diversity in Brazil due to its territorial extension and continental features [27]. In the present study, we found that the Southeast and the Northeast macro-regions of Brazil had the greatest number of children younger than 2 years old who were hospitalised due to ACSC, but higher rates of avoidable hospitalisations occurred in the North. Although the majority of available studies indicated that the rates of avoidable hospitalisation were higher in Southeast cities, particularly when related to respiratory diseases, it is likely that the North is a large macro-region with barriers that prevent offering the population adequate primary care and, consequently, provide less protection against hospitalisations by ACSC. Thus, it is important to consider that nosographic conditions for hospitalisation depend on the location and the environment [33, 35, 37].

In the last decades, residents from the North and Northeast regions of Brazil have migrated to the Southeast because of the great economic development in that macro-region and the possibility of better living conditions for their families. In addition to the high migration rates, the hospital infrastructure in the Southeast has one of the greatest number of beds in Brazil (2.35 per 1000 inhabitants), which increases accessibility and, consequently, the likelihood of greater numbers of hospitalisation cases. Moreover, a physician’s behaviour can be influenced by the existence of available beds, which can also directly influence the hospital admission rate because doctors decide whether to admit a child for hospitalisation [31, 38, 39].

Male gender was found to be related to the risk of being hospitalised for ACSC, which is consistent with the literature; however, the use of ambulatory care was greater among females compared with males, and girls were more likely to be taken care by their mothers and/or guardians [7, 39].

Lower socioeconomic conditions present the same risk, as explained by Berkson’s paradox: poor children are hospitalised more frequently than rich children with equally severe episodes. Poor children tend to have poor sanitation and poor living conditions, which leads to more episodes of diarrhoea; at the same time, poor children’ tend to be exposed to poor indoor air quality and smoking parents who are less likely to purposefully avoid smoking around their children, leading to more episodes of respiratory diseases. In addition, the ABEP level considers parental education when classifying a population. Thus, classes D and E had fewer years of education, which leads to less information, including information about healthcare. Moreover, there is the influence of a physician’s judgment regarding which children require hospitalisation, as explained above. This decision is directly influenced by socioeconomic conditions because poor people usually lack ideal conditions to provide adequate treatment at home. Together, these factors lead to higher hospitalisations rates for lower socioeconomic status’ children [11, 31, 40]. According to a Canadian cohort study, children born to low-income families had higher rate of hospitalisation during the first year of life, and this association usually persists for up to 9 years after birth, which shows the long-term consequences of social inequalities [32].

Children with mothers younger than 20 years old had a greater risk of avoidable hospitalisation. However, data in the literature is contradictory. Regarding hospitalisation for acute respiratory diseases and diarrhoea, the mother’s age has not been demonstrated as a risk factor in some studies [34, 36]. However, Fuchs, Victora and Fachel (1996) have demonstrated that an adolescent mother’s children have a greater risk of being hospitalised for diarrhoea because they are less prepared to manage this situation. This supposed unpreparedness is related to the change in social representations when a daughter who is entirely dependent suddenly becomes a mother who must provide and care for someone else. Although an adolescent is physically ready to become a mother, she is generally not socially, psychologically and economically ready because the Brazilian society has not favoured this competence, and adolescent pregnancy has been viewed as a socially vulnerable condition [41, 42].

The fact that a child was not breastfed in the first hour of life also increases the risk for avoidable hospitalisation. Breastfeeding in the first hour of life corresponds to the fourth step of the Baby-Friendly Hospital Initiative (BFHI), working as a predictor for exclusive and total breastfeeding. Adequate breastfeeding practices can decrease infant morbimortality and reduce the occurrence of infectious diseases, such as diarrhoea and pneumonia. Supposedly, the action of breastfeeding in the delivery room is linked to a greater quality of child feeding in the first year of life and a lower risk of being hospitalised because of ACSC. In addition, breastfeeding in the first hour can be influenced by the healthcare provided before and during labour, which means that poor prenatal visits can lead to a mother’s lack of information concerning breastfeeding and its benefits. Additionally, depending on the routines and quality of the hospital staff, this practice may be more or less valued, leading the health care professionals to encourage the mother (or not) to suckle her child in the immediate postpartum period [43–45]. This finding indicates how one small action can sometimes protect children from future adverse events, but it is most likely related more to the joint phenomena rather than to the fact itself.

Neonatal hospitalisation was also associated with a greater risk of future avoidable hospitalisations, after accounting for the healthcare provided to pregnant women and their newborns in the antepartum period, during labour and right after birth. This critical period helps to determine good health in the first 2 years of life, during which the main reasons for hospitalisation (e.g., low birth weight, premature birth, asphyxia and infectious diseases) may delay a child’s growth and development and result in greater susceptibility to infectious diseases, such as pneumonia and diarrhoea. Because children hospitalised in the neonatal period usually have lower immunity and worse general health than children who are not hospitalised during this period, the risk of being hospitalised during infancy is greater [46, 47].

The identification of situations in which children were more likely to experience avoidable hospitalisation gain greater validity with the inclusion of other factors that potentially influence hospitalisations in the multivariate analysis, thereby providing the broad perspective necessary to identify events triggered by multiple risk factors [48]. Thus, the effects that were shown to be significant after controlling for other variables in the multifactorial model included child age, residence area and macro-region.

Considering the measurement of the effectiveness of primary care and the development of public health strategies to decrease avoidable hospitalisations, it is important evaluate mechanisms related to access to healthcare as number of hospitals and physicians, the scope of provided care including the coordination between different levels of attention and, particularly, social inequalities such as socioeconomic, demographic and epidemiological characteristics as those highlighted above [5].

This study was performed using rigorous data gathering and analysis methods; however, including newborns in the sample may have affected the comparison of our results with studies that only included infants. Moreover, the age group chosen for this research has not usually been the focus of studies investigating hospitalisation rates and associated factors. These children are usually physically immature and, consequently, their health statuses are more vulnerable, placing them (as a group) at risk of hospitalisation [11].

Once the NDHS—2006 is based on a national representative sample for Brazilian infants and is similar to other research based on demographic health surveys, this study has performed all statistical procedures with the expansion technique of complex samples to ensure the data legitimacy [49].

Note that a possible limitation of this study is that hospitalisation rates may not reflect the real necessity of hospitalisation because they can be influenced by the population’s accessibility to care, physician judgments, quantity of available beds, and health service infrastructure. Hence, it is important to use this indicator sparingly, preferably by considering these variables in the analysis [31]. In addition, the data are reflective of the mother’s capacity to recall events in the past 12 months, which may have limited the accuracy of the information.

Furthermore, we were limited to using variables contained in the NDHS—2006 because the analysis was conducted using a pre-existing dataset. The only three conditions related to hospitalisation that were specified were diarrhoea, pneumonia and bronchitis, which limit knowledge of the cause of hospitalisation.

Although these limitations potentially lead to misinterpretation, it is unlikely that their extension has affected or depreciated the meaning and the validity of the estimated prevalence ratios. Once the NDHS - 2006 is the main set of data available in Brazil, the results should be useful for health professionals and managers aware of the strengths and limitations of that information as happen with national health surveys based on mothers’ interviews [50].

## Conclusions

This study highlights the fact that strategies to improve children’s health in Brazil by controlling avoidable hospitalisations should include health education and promotion in all residence areas and geopolitical macro-regions, particularly for young mothers from low socioeconomic backgrounds. Lifestyle quality improvements based on effective access to primary healthcare, particularly prenatal and paediatric care, including the prevention of neonatal problems and the stimulation of adequate breastfeeding, may be essential goals for communities and state authorities.

Because these associations do not represent causality but instead identify risk factors, health professionals should develop a special outpatient care schedule for children with Fig. 3 characteristics to avoid avoidable hospitalisations.

Furthermore, the characterisation of avoidable hospitalisation in children as an undesirable condition, particularly in the first years of life, indicates that health managers should develop and execute more direct, focused and integrated strategies involving all public health and education systems to effectively prevent such hospitalisations.

In addition, these results reinforce the importance of the impact of gastrointestinal and respiratory diseases that cause children to have avoidable hospitalisations. The development of strategies to control these morbidities could substantially decrease the hospitalisation costs, thereby improving resource allocation, particularly in developing countries such as Brazil.

Finally, given the multiple factors involved in avoidable hospitalisations in children, additional qualitative or quantitative studies that examine community needs are required to determine potential opportunities for health and education interventions*.*
